# Factors associated with glycemic control in community-dwelling elderly individuals with type 2 diabetes mellitus in Zhejiang, China: a cross-sectional study

**DOI:** 10.1186/s12902-019-0384-1

**Published:** 2019-06-06

**Authors:** Hong-Ting Zhu, Min Yu, Hao Hu, Qing-Fang He, Jin Pan, Ru-Ying Hu

**Affiliations:** 1 Yongkang Municipal Center for Disease Control and Prevention, Jinghua, Zhejiang, 321300 China; 2 Department of Occupational Diseases, Zhejiang Academy of Medical Sciences, Zhejiang, 310013 Hangzhou China; 3 Yongkang Municipal Center for Disease Control and Prevention, Jinghua, Zhejiang, 321300 China; 4 Department of NCDs Control and Prevention, Zhejiang Provincial Center for Disease Control and Prevention, No. 3399, Binsheng Road, Zhejiang, 310051 Hangzhou China

**Keywords:** Adiposity, Elders, Glycemic control, The product of triglyceride and glucose, Type 2 diabetes mellitus

## Abstract

**Background:**

Although exercise seems to be beneficial for type 2 diabetes mellitus (T2DM) patients, there is limited research elucidating the optimal accessible indices of adiposity and insulin resistance for identifying elderly T2DM patients with poor glycemic control, which could be improved by performing regular exercise.

**Methods:**

A community-based, cross-sectional study was conducted with 918 Chinese elderly individuals with T2DM in Zhejiang. Relevant risk factors for poor glycemic control, as determined using glycated haemoglobin A1c (HbA1c) > 7%, were explored using logistic regression analyses and included body mass index (BMI), waist circumference (WC), waist to height ratio (WHtR), fasting blood glucose (FBG), triglycerides (TGs), total cholesterol (TC), the product of fasting triglycerides and glucose (TyG), visceral adiposity index (VAI), lipid accumulation product (LAP), TyG-BMI, and TyG-WC. Comparisons of the risk factors’ ability to discriminate poor glycemic control as well as their optimal cutoff values were determined using receiver operating characteristic (ROC) analyses, and then the extent of poor glycemic control risk reduction through regular exercise was examined using multivariate logistic regression analyses.

**Results:**

The overall poor glycemic control rate was 49.3%. The factors associated with poor glycemic control included FBG > 3.869, TyG > 8.73, TyG-BMI > 222.45, and TyG-WC > 713.48 in logistic regression analyses. The optimal cutoff points of FBG, TyG, TyG-WC, and TyG-BMI in discriminating poor glycemic control were 7.38, 9.22, 813.33, and 227.77, and their corresponding areas under the ROC curves were 0.864(0.840–0.886), 0.684(0.653–0.714), 0.604(0.571–0.635), and 0.574(0.541–0.606), respectively. Occasional and regular exercise reduced the odds ratios (95% confidence interval) of poor glycemic control to 0.187 (0.063–0.557) and 0.183 (0.059–0.571) for subjects with TyG-WC > 813.33 (*p* = 0.008), to 0.349 (0.156–0.782) and 0.284 (0.123–0.652) for subjects with TyG > 9.22 (*p* = 0.011), and to 0.390 (0.175–0.869) and 0.300(0.130–0.688) for subjects with TyG-BMI > 227.77 (*p* = 0.017), respectively, after adjusting for multiple confounding factors.

**Conclusion:**

Among elderly individuals with T2DM, poor glycemic control risk might be identified using indices calculated from FBG, TG, BMI, and WC measurements, which are indicative of adiposity and insulin resistance. TyG-WC seems to be an accessible and useful indicator to identify which elderly T2DM patients would benefit from performing regular exercise to achieve good glycemic control.

**Electronic supplementary material:**

The online version of this article (10.1186/s12902-019-0384-1) contains supplementary material, which is available to authorized users.

## Background

Diabetes is one of the top 30 causes of years lived with disability, is affecting the elderly population and is becoming an important health issue in ageing global population [1]. In China, almost 60% of middle-aged and elderly population had prediabetes or diabetes in 2011–2012 [2], which will likely compromise the 33% premature mortality reduction goal between 2013 and 2030 even if all risk factors are attenuated [3]. Furthermore, diabetes-related cost accounts for a great proportion of the overall healthcare costs in China [4]. Therefore, it is necessary to optimize accessible measurements to identify individuals with suboptimal diabetes management to intervene as early as possible in China.

Type 2 diabetes mellitus (T2DM) management achievement could be assessed using integrative control targets, including fasting blood glucose, glycated haemoglobin A1c (HbA1c), blood pressure (BP), and lipid profile [5, 6]. HbA1c tests require sufficient health budget allocation and might not be readily accessible for constant diabetes management, especially in developing countries [7]. Recently, the product of fasting triglycerides and glucose, TyG index, appeared to be a simple and useful estimator of metabolic abnormalities, insulin resistance, T2DM onset and T2DM-related complication risk [8–12]. Alternative convenient indices, based on anthropometric parameters and lipid profile, have also been introduced to determine their association with impaired metabolic function and poor glycemic control in individuals with T2DM [13–15]. Despite the overall promising role of TyG, adiposity indices and TyG-adiposity integrated indices in predicting T2DM development and progression risk, their efficiency and optimal cutoff point for discriminating T2DM patients with poor glycemic control remains to be explored, especially for elderly individuals.

Physical activity and exercise have been demonstrated to be beneficial for individuals with T2DM by optimally controlling metabolic risk factors for diabetes management [16, 17]. Exercise-mediated glucose metabolism improvement might be attributed to adiposity and insulin resistance reduction, although the effective strategy of exercise (e.g., to what extent) to regulate glycemic homeostasis in elderly diabetic patients remains largely unknown [18–20]. The age-sensitive nature of HbA1c during ageing prompts the need to establish cutoff points of adiposity index for identifying the elderly population with abnormal glucose metabolism and other metabolic risks [21, 22]. Impaired glucose metabolism and the presence of adiposity and dyslipidemia might be related to insulin resistance, which contributes to physical inactivity for elderly individuals living in the community [23]. However, comprehensive knowledge of exercise, glycemic control, adiposity and insulin resistance remains to be established for elderly individuals with T2DM. Therefore, it would be interesting to quantify the effects of exercise frequency on reducing poor glycemic control risk of elderly T2DM patients as stratified by TyG or adiposity indices and to provide additional evidence for selecting exercise strategy to manage T2DM in elderly individuals with suboptimal glycemic control achievement.

This investigation aims to estimate the ability of TyG and other related metabolic risk factors to classify glycemic control status and to determine the appropriate index to discriminate elderly individuals with T2DM who are suitable candidates for regular exercise to improve glycemic control at the community level in Yongkang, Zhejiang, China.

## Methods

### Study participants

Data for this cross-sectional, observational study were retrieved from a local diabetes management system in Yongkang, Zhejiang, China. Subjects were residents aged ≥60 years and diagnosed with T2DM, and completed survey in March/April 2017. The procedure of this investigation is in accordance with the principles of the Declaration of Helsinki and is approved by the internal ethical review board of Zhejiang Provincial Center for Disease Control and Prevention, followed by acquiring informed consent from all participants.

### Parameters and measurements

The measurements of peripheral blood specimens retrieved for this study include circulation lipids profile, fasting blood glucose (FBG), and HbA1c. Circulation lipids profile includes triglycerides (TGs), total cholesterol (TC), low-density lipoprotein cholesterol (LDL-C), and high-density lipoprotein cholesterol (HDL-C). TGs and TC levels (mmol/L) were determined using glycerol phosphate oxidase-*p*-aminophenazone assay and cholesterol oxidase-*p*-aminophenazone assay, respectively, while LDL-C and HDL-C levels (mmol/L) were measured using the clearance method with LDL-C Direct Reagents (Siemens Healthcare Diagnostics Inc) and Direct HDL-C Reagents (Siemens Healthcare Diagnostics Inc) in ARCHITECT c16000 autoanalyzer (Abbott, Shanghai, China). FBG levels (mmol/L) were measured using hexokinase method in ARCHITECT c16000 autoanalyzer (Abbott, Shanghai, China). HbA1c levels (%) were determined using high-performance liquid chromatography method in MQ-2000PT autoanalyzer (Medconn Technology, Shanghai, China).

Complications were recorded and categorized into microvascular, macrovascular, and other comorbidities as previously described [24]. There were 28 subjects with microvascular comorbidities (6 with nephropathy, 6 with retinopathy, and 16 with neuropathy), 70 subjects with macrovascular comorbidities (3 with congestive heart failure, 28 with myocardial infarction, and 39 with stroke), and 1 subject with other complications (hypoglycemia).

The indices collected for the current investigation include anthropometric parameters, questionnaire items, and BP records. The anthropometric parameters consisted of height in centimeters (cm), weight in kilograms (kg), and waist circumference (WC) in cm. In addition, body mass index (BMI) = weight /height^2^. WC-to-height ratio (WHtR) was determined as WC/height. The questionnaire items from face-to-face interviews included income status (low, medium, high), duration of diabetes (in years), tobacco smoking (yes or no), alcohol drinking (yes or no), anti-diabetic medication (yes or no), medication adherence (poor or good), exercise (no, occasional, regular), and self-reported blood glucose monitoring (yes or no). Good medication adherence referred to taking anti-diabetic medication at the appropriate dose at the time advised by the doctor. Otherwise, poor medication adherence was recorded. Regular exercise referred to exercise performed > 30 min/day and > 3 days/week, indicating that diabetic participants maintained acceptable physical activity. Occasional exercise referred to exercise maintained to some extent but that falls outside of the regular exercise criteria mentioned above. No exercise referred to scarce physical activity and no conscious of effort towards exercise maintenance. BP recordings included diastolic blood pressure (DBP) in mmHg and systolic blood pressure (SBP) in mmHg.

Visceral adiposity index (VAI), lipid accumulation product (LAP), TyG, and TyG-related index was calculated using the following formulae as previously described [13, 25]: VAI _male_ = (WC/(39.68 + (1.88 × BMI))) × (TG/1.03) × (1.31/HDL-C); VAI_female_ = (WC/(36.58 + (1.89 × BMI))) × (TG/0.81) × (1.52/HDL-C); LAP_male_ = (WC − 65) × TG; LAP_female_ = (WC − 58) × TG; TyG = Ln(TG × FPG/2); TyG-BMI = TyG × BMI; TyG-WC = TyG × WC.

### Definitions of glycemic control target, anthropometric variables, and hypertension

For glycemic control evaluation, HbA1c ≤ 7% is recognized as optimal glycemic control, while HbA1c > 7% is considered as poor glycemic control [26, 27]. HbA1c at 7% is moderate glycemic control target for elderly individuals with T2DM [28]. For anthropometric indices associated with metabolic risk, sex-specific cutoff values of BMI, WC, and WHtR are 24.12 kg/m^2^, 83.5 cm and 0.51 for males and 23.53 kg/m^2^, 77.5 cm and 0.49 for females, respectively, as derived from the Chinese elderly population [21]. Furthermore, sex-specific cut-off values of VAI and LAP associated with impaired glucose metabolism are 2.25 and 36.96 for Chinese males and 2.5 and 37.84 for Chinese females, respectively [29]. Hypertension is defined as SBP ≥ 140 mmHg and/or DBP ≥ 90 mmHg.

### Statistical analysis

Quantitative data are reported as the mean with standard deviation or as the median with the 25th and 75th percentiles and were compared using independent t-tests or Mann-Whitney U tests depending on the normality of variables. Categorical variables were expressed as percentages and compared by Chi-square test or Fisher’s exact test where applicable. Correlation coefficients between two variables were determined using partial correlation analysis. Odds ratio (OR) with 95% confidence interval (CI) was calculated using binary logistic regression analysis to examine the effects of variables on glycemic control. The area under the receiver-operating characteristic (ROC) curve of the indicated variables was calculated with 95% CI, and the optimal cutoff point of variables for predicting poor glycemic control risk was determined according to the best Youden index (sensitivity+specificity-1). All statistical analyses were performed using IBM® SPSS® Statistics software (version 22.0) and MedCalc (version 18.11) and two-sided *p* value < 0.05 was considered statistically significant.

## Results

Among the 918 diabetic subjects recruited here, their mean age, BMI, and WC were 69.40 ± 6.09 years, 24.36 ± 3.08 kg/m^2^, and 85.06 ± 9.03 cm, respectively. In addition, their mean glucose, HbA1c, TG, TC, LDL, HDL, SBP and DBP levels were 7.76 ± 2.39 mmol/L, 7.35 ± 1.47%, 1.91 ± 1.45 mmol/L, 5.06 ± 1.07 mmol/L, 2.50 ± 0.79 mmol/L, 1.19 ± 0.26 mmol/L, 133.26 ± 13.27 mmHg, and 77.59 ± 7.78 mmHg, respectively. The majority of subjects were diagnosed with T2DM in the last 5 years (43.4%), reached the glycemic control target (HbA1c ≤ 7%) (50.7%), were without complications (89.2%), received anti-diabetic medication (85.3%), were females (57.0%), were non-drinkers (85.7%), and were non-smokers (90.1%), respectively. Additionally, the complication classifications stratified by diabetes duration showed that the microvascular, macrovascular, and other comorbidity percentage was 3% (12/398), 8.3% (33/398), and 0% (0/398) in T2DM subjects diagnosed in the last 5 years; 3.1% (12/381), 8.4% (36/453), and 0.3% (1/381) in T2DM subjects diagnosed in the last 6–10 years; and 2.9% (4/139), 3.6% (5/139), and 0% (0/139) in T2DM subjects diagnosed more than 10 years ago, respectively (*p* = 0.509).

The characteristics of the 918 diabetic subjects stratified by the HbA1c cutoff point (≤7% vs. > 7%) are shown in Table 1. Median duration of diabetes, FBG, HbA1c, TC, TyG, TyG-BMI, and TyG-WC were significantly higher in subjects with HbA1c > 7% than in the other subjects, and the proportion of individuals with a longer diabetes history (more than 6 years), anti-diabetic medication utilization and exercise deficit was also greater. However, there were no significant differences in age, sex, smoking status, alcohol drinking status, income status, self-blood glucose monitoring, medication adherence, complication classifications, height, weight, WC, BMI, WHtR, TG, LDL-C, HDL-C, VAI, LAP, or BP between subjects with HbA1c > 7% and ≤ 7%.

**Table 1:** Characteristics of diabetic subjects with HbA1c ≤7.0 and > 7.0%

	≤7.0% (*n* = 465)	> 7.0% (*n* = 453)	*p* value
Age, years	69 (65–73)	69 (65–73)	0.991
Male sex, n, %	205, 44.1%	190, 41.9%	0.512^a^
Tobacco smoking n, %	46, 9.9%	45, 9.9%	0.983^a^
Alcohol drinking, n, %	62, 13.3%	69, 15.2%	0.411^a^
Income status, n, %			
no data	159, 34.2%	161, 35.5%	
low	20, 4.3%	22, 4.9%	
medium	261, 56.1%	259, 57.2%	
High	25, 5.4%	11, 2.4%	0.145^b^
Height, cm	160 (155–165)	159 (154–164)	0.112
Weight, kg	62 (55–70)	61 (55–68)	0.194
Waist circumference, cm	85 (79–90)	85 (80–90)	0.666
BMI, kg/m^2^	24.44 (22.25–26.31)	24.10 (22.21–26.12)	0.498
WHtR	0.53 (0.50–0.56)	0.53 (0.50–0.57)	0.304
Duration of diabetes, years	6 (4–8)	7 (4–10)	< 0.001
Duration of diabetes, n, %			
in last 5 years	230, 49.5%	168, 37.1%	
6–10 years	184, 39.6%	197, 43.5%	
more than 10 years	51, 10.9%	88, 19.4%	< 0.001^a^
Fasting blood glucose, mmol/L	6.36 (5.68–7.08)	8.51 (7.39–10.35)	< 0.001
HbA1c, %	6.30 (5.90–6.70)	8.10 (7.50–9.10)	< 0.001
TC, mmol/L	4.91 (4.28–5.62)	5.06 (4.42–5.87)	0.014
TG, mmol/L	1.52 (1.11–2.13)	1.59 (1.13–2.29)	0.104
LDL-C, mmol/L	2.46 ± 0.75	2.54 ± 0.82	0.150
HDL-C, mmol/L	1.17 (1.02–1.34)	1.16 (1.00–1.34)	0.663
TyG	8.94(8.57–9.31)	9.30(8.93–9.75)	< 0.001
TyG-BMI	218.53(198.27–241.08)	225.88(204.68–250.18)	< 0.001
TyG-WC	758.76(693.72–819.11)	788.69(729.66–857.42)	< 0.001
VAI	2.13(1.40–3.10)	2.30(1.37–3.55)	0.100
LAP	35.47(23.67–51.73)	38.97(22.69–58.23)	0.085
DBP, mmHg	78 (70–82)	79 (72–80)	0.926
SBP, mmHg	130 (126–138)	130 (126–138)	0.998
Hypertension, n, %	105, 22.6%	94, 20.8%	0.501^a^
Complications, n, %			
No	414, 89%	405, 89.4%	
microvascular	16, 3.4%	12, 2.6%	
macrovascular	34, 7.3%	36, 7.9%	
Other	1, 0.2%	0	0.666
Anti-diabetic medication, n, %	382, 82.2%	401, 88.5%	0.006^a^
Medication adherence, n, %			
no data	0	2, 0.5%	
Poor	15, 3.9%	18, 4.5%	
Good	367, 96.1%	381, 95.0%	0.534^b^
Exercise			
no data	1, 0.2%	0	
No	33, 7.1%	62, 13.7%	
occasional	253, 54.4%	244, 53.9%	
Regular	178, 38.3%	147, 32.4%	0.003^b^
Self-blood glucose monitoring, n, %	111, 23.9%	105, 23.2%	0.805^a^

Data are presented as mean with standard deviation or median with 25th to 75th percentile for continues variables depending on distribution of each variable and number with percent for categorize variables; *p*-value is determined using t-test for continues variables while using chi-square test (^a^) or Fisher’s exact test (^b^) for categorize variables

*BMI* body mass index, *WHtR* waist: height ratio, *HbA1c* glycated haemoglobin A1c, *TC* total cholesterol, *TG* triglycerides; *LDL-C* low-density lipoprotein cholesterol, *HDL-C* high-density lipoprotein cholesterol, *DBP* diastolic blood pressure, *SBP* systolic blood pressure

The correlations between HbA1c and other metabolic risk factors are described in Table 2. HbA1c was significantly positively correlated with FBG (r = 0.765, *p* < 0.001), TC (r = 0.107, *p* = 0.009), TG (r = 0.105, *p* = 0.011), TyG (r = 0.383, *p* < 0.001), TyG-BMI (r = 0.152, *p* < 0.001), TyG-WC (r = 0.205, *p* < 0.001), VAI (r = 0.086, *p* = 0.037), and LAP (r = 0.084, *p* = 0.041). However, no significant association between HbA1c and HDL-C, LDL-C, BMI, WC, and WHtR was found.

**Table 2:** Correlations between HbA1c and FBG, lipids and metabolic risk factors in type 2 diabetic subjects

		FBG	HDL-C	LDL-C	TC	TG	BMI	WC	WHtR	TyG	TyG-BMI	TyG-WC	VAI	LAP
HbA1c	*r*	0.765	0.058	0.033	0.107	0.105	−0.029	−0.011	0.004	0.383	0.152	0.205	0.086	0.084
	*p*	< 0.001	0.160	0.432	0.009	0.011	0.480	0.784	0.916	< 0.001	< 0.001	< 0.001	0.037	0.041

*HbA1c* glycated haemoglobin A1c, *FBG* fasting blood glucose, *HDL-C* high-density lipoprotein cholesterol, *LDL-C* low-density lipoprotein cholesterol, *TC* total cholesterol, *TG* triglycerides, *BMI* body mass index, *WC* waist circumstance, *WHtR* waist: height ratio, *TyG* the product of triglycerides and fasting blood glucose, *VAI* visceral adiposity index, *LAP* lipid accumulation product. Correlations were determined using partial correlation analysis with adjustment of age, gender, income status, exercise, anti-diabetic medication, self-blood glucose test, alcohol drinking, tobacco smoking, duration of diabetes, complications and hypertension

The potential risk factors associated with HbA1c > 7.0% included TC > 5.71 mmol/L, FBG > 3.869 mmol/L, TyG > 8.73, TyG-BMI > 222.45, and TyG-WC > 713.48 in univariate logistic regression analyses (Table 3, Additional file 1). All these factors were also independently associated with HbA1c > 7.0%, except for TC, according to the results of multivariate logistic regression (Table 3). Moreover, ROC curve analysis showed the value of FBG, TyG, TyG-WC, TyG-BMI, and TC could classify individuals with HbA1c > 7.0%, although their efficiency differed. The AUCs (95% CI) were 0.864 (0.840–0.886) for FBG, 0.684 (0.653–0.714) for TyG, 0.604 (0.571–0.635) for TyG-WC, 0.574 (0.541–0.606) for TyG-BMI, and 0.547 (0.514–0.580) for TC (Table 4).

**Table 3:** Effects of variables on risk of HbA1c > 7.0% in diabetic subjects

	odds Ratio (95%CI)^a^	*p* value	odds Ratio (95%CI)^b^	*p* value
BMI				
Low (reference)	1.0		1.0	
High	0.779 (0.600–1.011)	0.060	0.879 (0.629–1.227)	0.448
WC				
Low (reference)	1.0		1.0	
High	1.078 (0.805–1.444)	0.613	1.189 (0.822–1.721)	0.358
WHtR				
Low (reference)	1.0		1.0	
High	1.262 (0.951–1.675)	0.107	1.335 (0.925–1.926)	0.123
LAP				
Low (reference)	1.0		1.0	
High	1.283(0.990–1.664)	0.060	1.191(0.851–1.667)	0.308
VAI				
Low (reference)	1.0		1.0	
High	1.247(0.960–1.619)	0.098	1.300(0.927–1.824)	0.128
TC				
< 4.35 (reference)	1.0		1.0	
4.35–4.97	1.127 (0.781–1.627)	0.523	0.988 (0.620–1.604)	0.992
4.97–5.71	1.366 (0.947–1.971)	0.095	1.216 (0.751–1.969)	0.427
> 5.71	1.547 (1.071–2.234)	0.020	1.209 (0.743–1.989)	0.438
TG				
< 1.11 (reference)	1.0		1.0	
1.11–1.55	0.948 (0.658–1.367)	0.776	1.227 (0.759–1.983)	0.404
1.55–2.18	1.139 (0.790–1.643)	0.485	1.274 (0.788–2.060)	0.323
> 2.18	1.179 (0.818–1.700)	0.376	1.393 (0.866–2.241)	0.172
LAP				
< 23.11 (reference)	1.0		1.0	
23.11–37.26	0.755 (0.522–1.091)	0.134	0.970 (0.603–1.561)	0.900
37.26–56.08	1.018 (0.705–1.468)	0.926	1.030 (0.634–1.675)	0.904
> 56.08	1.141 (0.790–1.647)	0.482	1.242 (0.769–2.007)	0.376
VAI				
< 1.38 (reference)	1.0		1.0	
1.38–2.20	0.758 (0.524–1.095)	0.139	0.948 (0.585–1.535)	0.827
2.20–3.36	0.932 (0.645–1.347)	0.709	1.175 (0.722–1.912)	0.517
> 3.36	1.290 (0.892–1.866)	0.176	1.268 (0.775–2.076)	0.344
FBG				
< 6.19 (reference)	1.0		1.0	
6.19–7.19	3.865 (2.347–6.364)	< 0.001	5.270 (2.601–10.681)	< 0.001
7.19–8.68	13.892 (8.475–22.772)	< 0.001	16.634 (8.225–33.637)	< 0.001
> 8.68	95.667 (50.662–180.650)	< 0.001	111.656 (47.765–261.008)	< 0.001
TyG				
< 8.73 (reference)	1.0		1.0	
8.73–9.14	2.024 (1.378–2.972)	< 0.001	2.301 (1.382–3.833)	0.001
9.14–9.51	2.558 (1.740–3.761)	< 0.001	3.073 (1.853–5.096)	< 0.001
> 9.51	6.411 (4.271–9.623)	< 0.001	6.698 (3.958–11.338)	< 0.001
TyG-BMI				
< 200.64 (reference)	1.0		1.0	
200.64–222.45	1.313 (0.908–1.898)	0.148	1.301 (0.806–2.100)	0.282
222.45–246.28	1.549 (1.071–2.241)	0.020	1.871 (1.166–3.001)	0.009
> 246.28	2.004 (1.382–2.906)	< 0.001	2.098 (1.306–3.370)	0.002
TyG-WC				
< 713.48 (reference)	1.0		1.0	
713.48–772.10	1.559 (1.074–2.261)	0.019	2.122 (1.294–3.480)	0.003
772.10–840.36	1.642 (1.132–2.382)	0.009	1.848 (1.137–3.004)	0.013
> 840.36	2.837 (1.943–4.141)	< 0.001	3.416 (2.069–5.642)	< 0.001

*HbA1c* glycated haemoglobin A1c, *BMI* body mass index, *WC* waist circumstance, *WHtR* waist: height ratio, *TC* total cholesterol, *TG* triglycerides, *LAP* lipid accumulation product, *VAI* visceral adiposity index, *TyG* the product of triglycerides and fasting blood glucose, *CI* confidence interval

^a^crude odds ratio determined using univariate logistic regression (enter method)

^b^odds ratio determined using multivariate logistic regression (enter method) with adjustment of age, gender, alcohol drinking, tobacco smoking, duration of diabetes, income status, exercise, anti-diabetic medication, self-blood glucose monitoring, complications and hypertension. The cut-off level of BMI, WC, WHtR, VAI, and LAP is 24.12 kg/m^2^, 83.5 cm, 0.51, 2.25, and 36.96for males, while 23.53 kg/m^2^, 77.5 cm, 0.49, 2.5, and 37.84 for females, respectively

**Table 4:** Cut-off value and area under curve (AUC) of variables for prediction of HbA1c > 7.0% risk in diabetic subjects

	Cut-off value	Sensitivity	Specificity	Youden Index	AUC(95%CI)	*p* value^a^	*p* value
FBG	7.38	0.7506	0.8387	0.5893	0.864(0.840–0.886)	< 0.001	< 0.022^b^
TyG	9.22	0.5762	0.6989	0.2751	0.684(0.653–0.714)	< 0.001	< 0.022^b^
TyG-WC	813.33	0.4336	0.7376	0.1713	0.604(0.571–0.635)	< 0.001	< 0.022^b^
TyG-BMI	227.77	0.4790	0.6366	0.1156	0.574(0.541–0.606)	< 0.001	< 0.022^c^
TC	5.98	0.2185	0.8753	0.0938	0.547(0.514–0.580)	0.013	0.283^d^

*HbA1c* glycated haemoglobin A1c, *FBG* fasting blood glucose, *TyG* the product of triglycerides and fasting blood glucose, *TC* total cholesterol, *TG* triglycerides, *WC* waist circumstance, *BMI* body mass index, *CI* confidence interval

^a^AUC of each variable compared with 0.5

^b^AUC of the indicated variable compared with all the other variables

^c^AUC of TyG-BMI compared with AUC of all the other variables except for TC

^d^AUC of TC compared with AUC of TyG-BMI

Diabetic subjects with poor glycemic control (HbA1c > 7.0%) were screened using the optimal cutoff value of indices as described in Table 4, followed by exploring the effect of exercise in reducing risk of HbA1c > 7.0%. As shown in Table 5 with Additional file 1, an increase in exercise frequency produced a significant decrease in HbA1c > 7.0% risk for diabetic subjects with TyG > 9.22 (*p* = 0.011), TyG-WC > 813.33 (*p* = 0.008), and TyG-BMI > 227.77 (*p* = 0.017). For instance, among diabetic subjects with TyG-WC > 813.33, their OR (95% CI) of HbA1c > 7.0% risk was reduced to 0.187 (0.063–0.557) for subjects taking occasional exercise and further decreased to 0.183 (0.059–0.571) for subjects performing regular exercise compared to subjects without exercise. However, there was no significant impact of exercise on HbA1c > 7.0% risk reduction for diabetic subjects with FBG > 7.38 or TC > 5.98.

**Table 5:** Effects of exercise on reducing HbA1c > 7.0% risk in diabetic subjects with indicated metabolic risk factor

	FBG > 7.38	*p*	TyG > 9.22	*p*	TyG-WC > 813.33	*p*	TyG-BMI > 227.77	*p*	TC > 5.98	*p*
	OR, 95%CI^a^		OR, 95%CI^a^		OR, 95%CI^a^		OR, 95%CI^a^		OR, 95%CI^a^	
Exercise										
no (reference)	1.00		1.00		1.00		1.00		1.00	
occasional	0.411(0.157–1.072)		0.349 (0.156–0.782)		0.187 (0.063–0.557)		0.390 (0.175–0.869)		0.311 (0.083–1.169)	
regular	0.438 (0.162–1.186)	0.180	0.284(0.123–0.652)	0.011	0.183 (0.059–0.571)	0.008	0.300 (0.130–0.688)	0.017	0.320 (0.093–1.102)	0.147

*HbA1c* glycated haemoglobin A1c, *FBG* fasting blood glucose, *TyG* the product of triglycerides and fasting blood glucose, *BMI* body mass index, *WC* waist circumstance, *TC* total cholesterol

^a^odds ratio with 95% confidence interval (OR, 95%CI) determined using multivariate logistic regression (enter method) with adjustment of age, gender, alcohol drinking, tobacco smoking, duration of diabetes, income status, anti-diabetic medication, self-blood glucose monitoring, complications and hypertension

## Discussion

The good glycemic control rate from 2010 to 2012 was reported to be 32.6% nationally with recent glycemic control improvement in the southern-eastern part of China. However, discrepancies still existed between provinces and the relevant rates ranged from 49.7% in Ningbo, Zhejiang to 58.5% in Xiamen, Fujian [26, 30–32]. The updated data reflecting increased awareness and progress of diabetes management at the community level are presented here and indicate acceptable glycemic control and lifestyle, availability of anti-diabetic medication with good adherence, and low incidence of complications at least for elderly individuals with T2DM in 2017 in Yongkang, Zhejiang, China. In addition, poor glycemic control risk could be classified using FBG > 7.38, TyG > 9.22, TyG-WC > 813.33, and TyG-BMI > 227.77 with FBG being the most robust index. Nevertheless, poor glycemic control risk reduction through occasional and regular exercise appears to be much more effective for elderly T2DM patients with TyG-WC > 813.33 compared to the others with TyG > 9.22 and TyG-BMI > 227.77, suggesting that TyG-WC might be an accessible and auxiliary measurement to advice elderly T2DM patients to perform exercise for diabetes management.

Previous cross-sectional studies reported that HbA1c positively correlates with TC, TG, and LDL-C among T2DM patients in Turkey and Saudi Arabia but inversely associates with HDL-C in Saudi Arabian T2DM patients [33, 34]. Baseline HDL-C correlated with insulin resistance, but not with HbA1c, although lower HDL-C suggested a greater and earlier need for glycemic control in T2DM patients from a prospective intervention study in Australia [35]. Positive correlations between HbA1c and TC and TG were also observed, but HbA1c was not associated with LDL-C or HDL-C in Chinese elderly individuals with T2DM in this cross-sectional study. The inconsistent trend of changes between LDL-C and HbA1c were also reported in previous meta-analysis data [36] and elsewhere [37, 38], appearing to be dependent on the presence of lipid-lowering medication and cardiovascular diseases among individuals with T2DM [39].

The association between general/ central obesity measurements (e.g., BMI and WC) and uncontrolled glycemic status differed depending on the ethnic population investigated [15, 40, 41]. Abdominal adiposity, as determined using WHtR, has been demonstrated to be a better obesity indicator for predicting future T2DM risk in a meta-analysis based on prospective studies [42]. Moreover, sex-specific WHtR cutoff point might be helpful for predicting individuals with elevated HbA1c and subjects taking glucose-lowering medication in black South African population [43] and for screening T2DM subjects in Chinese population [44]. Sex-specific cutoff points of WC, BMI, and WHtR for Chinese elderly individuals are available [21], but might not be indicators of predicting poor glycemic control risk for elderly T2DM patients as presented here for the first time. The benefits of incorporation of almonds in a well-balanced healthy diet for improved glycemic and lipid control might be explained by decreased WC and WHtR values, as observed in a 24-week intervention study with 50 Asian Indian T2DM patients [45]. Nevertheless, the modification of diet on the relationship between anthropometric parameter-based adiposity index (e.g., BMI, WC, and WHtR) and glycemic control in the Chinese elderly individuals with T2DM remains to be investigated, which might provide possible evidence to optimize diet choice for diabetes management.

Recently, the anthropometric and lipid measurements-based visceral adiposity parameters (e.g., VAI and LAP) became effective and accessible indicators of insulin resistance in nondiabetic subjects [25] and exhibited promising performance in screening metabolic syndrome among the T2DM population [13], but showed no improved ability to indicate T2DM development [46]. Clinically, glycemic control improvement was evident in T2DM patients after 12 months of liraglutide treatment, which effectively reduced the VAI value [47]. In this filed study, neither VAI nor LAP was a useful tool for evaluating glycemic control in T2DM population regardless of sex, although positive correlations between VAI and LAP and HbA1c existed. Sufficient treatment information was available for patients recruited previously [47], which was not the case for the subjects investigated here due to the unavailability of BP and lipid control-related treatment. Nevertheless, VAI and LAP themselves might reflect lipid profile to some extent, and TC and TG were not independent risk factors for poor glycemic control, as indicated here. Furthermore, BP was further adjusted in our multivariate regression analysis. Sophisticated techniques, which directly measure visceral fat thickness and adipose hypertrophy, have revealed the potential role of adiposity in assessing glucose, lipid and insulin resistance in T2DM patients [48, 49]. Therefore, phenotype-based visceral adiposity measurements (e.g., VAI and LAP) should be simultaneously determined, followed by evaluation of their performance in predicting glycemic control status in individuals with T2DM in future field investigation.

To date, the positive correlation between TyG and TyG-adiposity parameters and the homeostasis model assessment of insulin resistance indicates that these parameters can be employed to recognize insulin resistance and metabolic syndrome in the research field without definitive discussion of these parameters in T2DM primary care guideline for clinical practice [8, 25, 50–52]. Previous studies have shown the role of TyG in predicting T2DM development risk among nondiabetic individuals [53] and in evaluating coronary artery stenosis risk among hyperglycemic individuals with poor glycemic control [54]. Nevertheless, TyG seemed to be a less robust predictor of future T2DM risk than FBG but more robust than TG, especially in individuals with impaired fasting glucose [55]. TyG measurement is inexpensive and routinely available and represents a useful tool to assess long term glycemic in T2DM patients with good correlation with HbA1c and insulin resistance [56]. The current finding considered TyG an indicator of poor glycemic control among hyperglycemic individuals, although TyG’s efficiency appeared to be lower than FBG but higher than TG. TyG with adiposity status has been proposed to be a surrogate marker for the early identification of insulin resistance [25] and of prediabetes and diabetes in first-degree relatives of T2DM patients [10]. Incremental increase in both TyG and BMI indicated an increased 8-year cumulative incidence of T2DM but appeared to have no obvious superiority in predicting T2DM risk compared to TyG being used as a forecasting determinant [53]. TyG appeared to discriminate metabolic unhealthy individuals independent of incremental increases in BMI and WC [9]. Neither TyG-BMI nor TyG-WC exhibited improved efficiency in discriminating T2DM patients with poor glycemic control compared to FBG or TyG in this study. Based on the finding mentioned above, FBG might be the most convenient and robust measurement to determine the risk of T2DM development and poor glycemic control, followed by TyG, TyG-WC, and TyG-BMI, respectively.

Exercise and weight loss may put T2DM into remission if applied early after diagnosis [57]. However, there is a concern that severe metabolic dysregulation could reduce benefit from exercise and exercise tends to exhibit metabolic benefit for T2DM patients with relative stable and good glycemic control [58]. T2DM subjects might suffer from dysmetabolic environment, endothelial, cardiac and peripheral dysfunction, or have insufficient exercise, which are likely attributed to insulin resistance [59]. Insulin resistance might be estimated using adiposity parameters (e.g., BMI, WC) [60, 61] or glucose and lipid-based indices (e.g., TyG) [7, 62]. For T2DM patients with higher level of HbA1c, increased exercise frequency per week seems to be an important approach to achieve favorable health outcome [63]. Among the Chinese elderly individuals with T2DM investigated here, subjects with TyG > 9.22, TyG-WC > 813.33, and TyG-BMI > 227.77 might be much better candidates to perform exercise to reduce poor glycemic control risk, which was not the case for subjects with FBG > 7.38 or TC > 5.98. Central obesity reduction seems to exert primordial function in glycemic control and personal characteristics must be taken into consideration for the choice of the best exercise modality for each elderly T2DM patient [18]. Coincidently, either occasional or regular exercise tended to maximally improve glycemic control, especially for subjects with TyG-WC > 813.33, which appeared to have higher specificity in predicting poor glycemic control compared to TyG > 9.22 or TyG-BMI > 227.77. Accordingly, exercise might be beneficial for glycemic control, especially for elderly T2DM patients with suspicious insulin resistance and adiposity, but not for elders with only elevated FBG or TC. This might be tentatively explained by the fact that exercise could increase insulin sensitivity and reduce resting insulin levels and body fat, thereby changing metabolic adaptions of T2DM patients [17]. Moreover, exercise could also decrease WC, body mass, BMI and the required hypoglycemic drug dose alone with improved glycemic control, which provided great benefits for T2DM patients who are with inadequate glycemic control and are worrying about possible injection therapy (e.g., insulin) as revealed in a recent lifestyle intervention study [64].

There are some limitations in this study. First, multiple risk factors associated with poor glycemic control are identified in this cross-sectional study, but their predictive values for evaluating glycemic control remained unknown. Second, exercise frequency is a categorical variable not an absolute frequency or amount of exercise, providing preliminary information regarding the potential benefit of regular exercise on glycemic control events for certain groups of elderly individuals with T2DM, but no definitive exercise training intensity advice. Third, the findings may or may not be applicable for T2DM patients with unstable conditions because this study recruited residents with T2DM living in the community but not T2DM cases at the hospital. Furthermore, the findings appear to be ethnic-dependent, which should be further tested in other ethnic T2DM populations.

## Conclusions

The current data suggest that poor glycemic control risk might be screened using indices derived from FBG, TG, and anthropometric parameters indicative of adiposity and insulin resistance among elderly individuals with T2DM. Among them, TyG-WC seems to be an accessible and useful indicator to identify the most suitable elderly T2DM patients to perform regular exercise to achieve good glycemic control.

## Additional file

Additional file 1: β and S.E. of parameter corresponding to binary logistic regression results. (DOCX 27 kb)

## Data Availability

The data are within the manuscript.

## Abbreviations

BMI: body mass index
BP: blood pressure
CI: confidence interval
DBP: diastolic blood pressure
FBG: fasting blood glucose
HbA1c: glycated haemoglobin A1c
HDL-C: high-density lipoprotein cholesterol
LAP: lipid accumulation product
LDL-C: low-density lipoprotein cholesterol
ORs: odds ratios
ROC: receiver-operating characteristic
SBP: systolic blood pressure
T2DM: type 2 diabetes mellitus
TC: total cholesterol
TG: triglycerides
TyG: the product of triglycerides and glucose
VAI: visceral adiposity index
WC: waist circumference
WHtR: waist circumference to height ratio
