# Supplementary material for: Factors associated with glycemic control in community-dwelling elderly individuals with type 2 diabetes mellitus in Zhejiang, China: a cross-sectional study
Source: BMC Endocr Disord. 2019 Jun 6;19:57. doi: 10.1186/s12902-019-0384-1 (PMC6555723; doi:10.1186/s12902-019-0384-1)
Supplement: Supplementary file 1 — β and S.E. of parameter corresponding to binary logistic regression results. (DOCX 27 kb) [file 12902_2019_384_MOESM1_ESM.docx]

**Table S1. β and S.E. for parameter corresponding to binary logistic regression results in Table 3**

|  | |  | **Univariate** |  |  | | **Multivariate** | | | |  |  |
| --- | --- | --- | --- | --- | --- | --- | --- | --- | --- | --- | --- | --- |
|  | | **β** | **S.E** | ***p*** | **β** | | **S.E** | | | | ***p*** |  |
| **BMI** | |  |  |  |  | |  | | | |  |  |
| constant | | 0.113 | 0.099 | 0.256 | 1.342 | | 1.095 | | | | 0.221 |  |
| high | | -0.250 | 0.133 | 0.060 | -0.129 | | 0.171 | | | | 0.448 |  |
| **WC** | |  |  |  |  | |  | | | |  |  |
| constant | | -0.081 | 0.149 | 0.524 | 1.212 | | 1.099 | | | | 0.270 |  |
| high | | 0.075 | 0.149 | 0.613 | 0.173 | | 0.188 | | | | 0.358 |  |
| **WHtR** | |  |  |  |  | |  | | | |  |  |
| constant | | -0.189 | 0.121 | 0.118 | 1.135 | | 1.100 | | | | 0.302 |  |
| high | | 0.233 | 0.144 | 0.107 | 0.289 | | 0.187 | | | | 0.123 |  |
| **LAP** | |  |  |  |  | |  | | | |  |  |
| constant | | -0.148 | 0.094 | 0.113 | 1.637 | | 1.130 | | | | 0.147 |  |
| high | | 0.249 | 0.133 | 0.060 | 0.175 | | 0.171 | | | | 0.308 |  |
| **VAI**  constant  high | | -0.123  0.221 | 0.089  0.133 | 0.168  0.098 | 1.581  0.262 | | 1.133  0.173 | | | | 0.163  0.128 |  |
| **TC** | |  |  |  |  | |  | | | |  |  |
| constant | | -0.243 | 0.132 | 0.067 | 1.110 | | 1.118 | | | | 0.321 |  |
| 4.35-4.97 | | 0.120 | 0.187 | 0.523 | -0.002 | | 0.242 | | | | 0.992 |  |
| 4.97-5.71 | | 0.312 | 0.187 | 0.095 | 0.195 | | 0.246 | | | | 0.427 |  |
| >5.71 | | 0.436 | 0.188 | 0.020 | 0.195 | | 0.251 | | | | 0.438 |  |
| **TG** | |  |  |  |  | |  | | | |  |  |
| constant | | -0.086 | 0.131 | 0.512 | 1.119 | | 1.104 | | | | 0.311 |  |
| 1.11-1.55 | | -0.053 | 0.186 | 0.776 | 0.204 | | 0.245 | | | | 0.404 |  |
| 1.55-2.18 | | 0.130 | 0.187 | 0.485 | 0.242 | | 0.245 | | | | 0.323 |  |
| >2.18 | | 0.165 | 0.186 | 0.376 | 0.331 | | 0.243 | | | | 0.172 |  |
| **LAP** | |  |  |  |  | |  | | | |  |  |
| constant | | 0.009 | 0.132 | 0.947 | 1.264 | | 1.105 | | | | 0.253 |  |
| 23.11-37.26 | | -0.281 | 0.188 | 0.134 | -0.030 | | 0.243 | | | | 0.900 |  |
| 37.26-56.08 | | 0.017 | 0.187 | 0.926 | 0.030 | | 0.248 | | | | 0.904 |  |
| >56.08 | | 0.132 | 0.187 | 0.482 | 0.217 | | 0.245 | | | | 0.376 |  |
| **VAI** | |  |  |  |  | |  | | | |  |  |
| constant | | -0.026 | 0.132 | 0.844 | 1.188 | | 1.107 | | | | 0.283 |  |
| 1.38-2.20 | | -0.140 | 0.187 | 0.453 | -0.054 | | 0.246 | | | | 0.827 |  |
| 2.20-3.36 | | 0.001 | 0.187 | 0.999 | 0.161 | | 0.248 | | | | 0.517 |  |
| >3.36 | | 0.149 | 0.187 | 0.426 | 0.238 | | 0.251 | | | | 0.344 |  |
| **FBG** | |  |  |  |  | |  | | | |  |  |
| constant | | -2.104 | 0.212 | <0.001 | -1.616 | | 1.376 | | | | 0.240 |  |
| 6.19-7.19 | | 1.352 | 0.254 | <0.001 | 1.662 | | 0.360 | | | | <0.001 |  |
| 7.19-8.68 | | 2.631 | 0.252 | <0.001 | 2.811 | | 0.359 | | | | <0.001 |  |
| >8.68 | 4.561 | | 0.324 | <0.001 | 4.715 |  | | 0.433 | <0.001 | | | |
| **TyG** | |  |  |  |  | |  | | | |  |  |
| constant | | -0.895 | 0.145 | <0.001 | 0.493 | | 1.152 | | | | 0.669 |  |
| 8.73-9.14 | | 0.705 | 0.196 | <0.001 | 0.833 | | 0.260 | | | | 0.001 |  |
| 9.14-9.51 | | 0.939 | 0.197 | <0.001 | 1.123 | | 0.258 | | | | <0.001 |  |
| >9.51 | | 1.858 | 0.207 | <0.001 | 1.902 | | 0.269 | | | | <0.001 |  |
| **TyG-BMI** | |  |  |  |  | |  | | | |  |  |
| constant | | -0.377 | 0.134 | 0.005 | 0.962 | | 1.117 | | | | 0.389 |  |
| 200.64-222.45 | | 0.272 | 0.188 | 0.148 | 0.263 | | 0.244 | | | | 0.282 |  |
| 222.45-246.28 | | 0.438 | 0.188 | 0.020 | 0.626 | | 0.241 | | | | 0.009 |  |
| >246.28 | | 0.695 | 0.190 | <0.001 | 0.741 | | 0.242 | | | | 0.002 |  |
| **TyG-WC** | |  |  |  |  | |  | | | |  |  |
| constant | | -0.522 | 0.136 | <0.001 | 0.892 | | 1.124 | | | | 0.427 |  |
| 713.48-772.10 | | 0.444 | 0.190 | 0.019 | 0.752 | | 0.252 | | | | 0.003 |  |
| 772.10-840.36 | | 0.496 | 0.190 | 0.009 | 0.614 | | 0.248 | | | | 0.013 |  |
| >840.36 | | 1.043 | 0.193 | <0.001 | 1.229 | | 0.256 | | | <0.001 | |  |

**Table S2. β and S.E. for parameter corresponding to binary logistic regression results in Table 5**

|  | **FBG>7.38** | | | **TyG>9.22** | | | **TyG-WC>813.33** | | | **TyG-BMI>227.77** | | | **TC>5.98** | | |
| --- | --- | --- | --- | --- | --- | --- | --- | --- | --- | --- | --- | --- | --- | --- | --- |
|  | **β** | **S.E.** | ***p*** | **β** | **S.E.** | ***p*** | **β** | **S.E.** | ***p*** | **β** | **S.E.** | ***p*** | **β** | **S.E.** | ***p*** |
| **Exercise** |  |  |  |  |  |  |  |  |  |  |  |  |  |  |  |
| constant | 2.835 | 2.168 | 0.191 | 3.662 | 1.794 | 0.041 | 3.294 | 2.197 | 0.134 | 4.520 | 1.893 | 0.017 | 4.014 | 3.262 | 0.218 |
| occasional | -0.890 | 0.490 | 0.069 | -1.052 | 0.411 | 0.010 | -1.677 | 0.557 | 0.003 | -0.941 | 0.409 | 0.021 | -1.169 | 0.676 | 0.084 |
| regular | -0.826 | 0.508 | 0.104 | -1.260 | 0.425 | 0.003 | -1.698 | 0.580 | 0.003 | -1.205 | 0.424 | 0.005 | -1.139 | 0.631 | 0.071 |
